# Variable-Temperature Size Exclusion Chromatography for the Study of the Structural Changes in G-Quadruplex

**DOI:** 10.1155/2013/631875

**Published:** 2013-11-10

**Authors:** Sanae Benabou, Ramon Eritja, Raimundo Gargallo

**Affiliations:** ^1^Solution Equilibria and Chemometrics Group (Associate Unit UB-CSIC), Department of Analytical Chemistry, University of Barcelona, Diagonal 647, 08028 Barcelona, Spain; ^2^Institute of Advanced Chemistry of Catalonia (IQAC-CSIC), CIBER-BBN Networking Research Centre on Bioengineering, Biomaterials and Nanomedicine, Jordi Girona 18-26, 08034 Barcelona, Spain

## Abstract

The conformational equilibria of a guanine-rich sequence found at the promoter region of the human *c-kit* oncogene are studied by means of circular dichroism spectroscopy (CD) and variable-temperature size exclusion chromatography (SEC). It is shown that the wild sequence ckit21 exists as a mixture of monomeric and multimeric G-quadruplexes. Appropriate mutation of several bases in the wild sequence produces the shift from parallel to antiparallel G-quadruplex, as well as the disappearance of multimeric species. The shift from the antiparallel to the parallel conformation induced by temperature is reflected in both CD and SEC profiles.

## 1. Introduction

Guanine-rich regions can fold into complex structures known as G-quadruplexes, whose building block is the tetrad, an almost planar arrangement of four guanine bases bonded by hydrogen bonds [1]. Cations, such as Na^+^ or K^+^, are known to strongly stabilize such structures by intercalation in the middle or among the tetrads. Depending on the relative orientation of the guanine tracts, antiparallel, parallel, or mixed topologies can be found. The formation of G-quadruplexes in sequences corresponding to the telomeres and to the promoter regions of several oncogenes has been deeply studied *in vitro* by means of disparate instrumental techniques [2, 3]. Recently, the presence *in vivo* of these structures has been observed [4].

**Figure 1:**
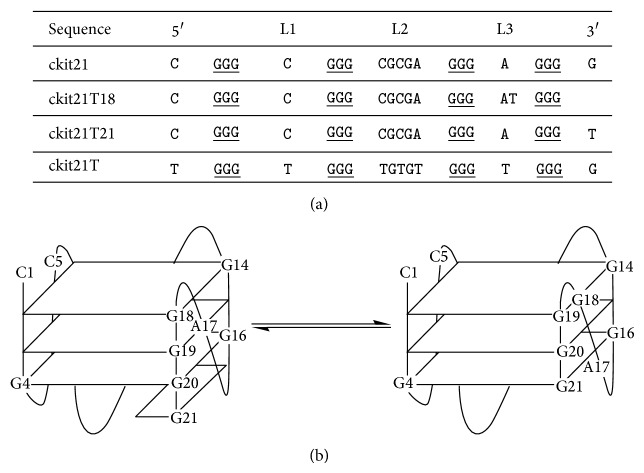
(a) Sequences studied in this work. (b) Dangling- to blunt-end equilibrium in ckit21.

In their pioneer work, Fernando et al. identified a G-quadruplex forming sequence upstream of the *c-kit* transcription initiation site [5]. The wild sequence (herein identified as ckit21, Figure 1) contains three tracts of three guanines and a fourth tract comprising four guanines. Based on CD, NMR, and UV-monitored melting experiments, the mutated sequence ckit21T18 was shown to be less heterogeneous than ckit21, whereas a homogeneous parallel G-quadruplex was proposed for the mutated sequence ckit21T21. In a later work, Hsu et al. studied the conformational space of ckit21 and ckit21T21 by means of NMR [6]. Their results confirmed that, in presence of a concentration of K^+^ around 100 mM, ckit21 existed as an ensemble of monomeric structures that share a parallel-stranded propeller-type conformation. Schematically, these structures produced two similar folding patterns named dangling-end and blunt-end (Figure 1). Hence, the sequence ckit21T18 should form the blunt-end structure, including a two-base third loop, whereas the sequence ckit21T21 should form the dangling-end structure, including a single-base reversal-loop. Almost simultaneously to the previous paper, the existence of a monomer-dimer equilibrium for the ckit21T21 sequence was also proposed [7]. Based on NMR measurements, it was suggested that concentrations of K^+^ ~100 mM shifted the equilibrium to the dimer species. Very recently, the polymorphism of the ckit21T21 sequence has been studied by means of size exclusion chromatography (SEC) [8]. At ~150 mM K^+^ and 200 mM DNA concentration, the existence of monomeric, dimeric, and tetrameric species in equilibrium was proposed. However, none of the recorded NMR spectra for the purified species matched the previously reported NMR data for that sequence. It seems that the formation of a particular quadruplex is dependent on many factors, not only on the sequence [9, 10].

In the present work, the effect of several mutations on the ckit21 sequence by means of CD and SEC measurements done at different temperatures was studied. The DNA concentration used for both measurements was around 2–5 *μ*M (the order of magnitude used in most of the spectroscopic measurements), and the K^+^ concentration was 150 mM. In addition to the three sequences already mentioned (ckit21, ckit21T18, and ckit21T21), a fourth sequence (ckit21T), where all cytosine and adenine bases were mutated to thymine, was studied. The results showed that the mutation in appropriate locations was clearly reflected into the chromatograms. The complete mutation in ckit21T produced a dramatic reduction of the conformational polymorphism shown by the wild sequence. Besides, variable-temperature HPLC has been already used to study the conformational equilibria of oligonucleotides [11]; to our knowledge this is the first report on the use of variable-temperature SEC to complement spectroscopic information related to the G-quadruplex structure.

## 2. Material and Methods

### 2.1. Reagents

DNA stock solutions were prepared in MilliQ water, stored at −20°C, and diluted to working concentrations in MilliQ water immediately before use. The composition of quadruplex stabilizing buffer (QSB) was 7 mM KH_2_PO_4_, 10 mM Na_2_HPO_4_, and 147 mM KCl pH 7.2. Oligonucleotide 5′-d[CG_3_CG_3_CGCGAG_3_AG_4_]-3′, designated as ckit21, was a G-quadruplex forming element in the promoter region of the human *c-kit* protooncogene. Oligonucleotide 5′-d[TG_3_TG_3_TGTGTG_3_TG_4_]-3′, designated as ckit21T, was a mutated sequence in which adenine and cytosine bases of the wild type ckit21 were systematically replaced with thymine. Two additional sequences ckit21T18 and ckit21T21 were designed to freeze the dangling-end to blunt-end equilibrium. These oligonucleotides were prepared as described elsewhere [13]. Oligonucleotide concentration was determined by UV absorbance measurements at 260 nm using calculated extinction coefficients and the nearest-neighbor method as implemented in OligoCalc webpage [14]. The integrity of DNA samples was checked by means of MS measurements. Oligonucleotides were annealed by raising the temperature to 90°C for 10 min and then cooling to room temperature overnight prior to melting or SEC measurements.

### 2.2. Instrumentation and Procedures

Absorbance and CD spectra were measured using an Agilent HP8453 photodiode array spectrophotometer and a Jasco J-810 spectropolarimeter, respectively. Both of them are equipped with stirrer and Peltier units mounted in the spindle of the thermoelectric cuvette holders. Melting curves were collected on the J-810 instrument. The concentration of DNA samples was around 3 *μ*M. Samples were heated at a linear temperature ramp of 0.3°C/min with data collection beginning at 25°C and ending at 95°C. A blank dataset taken from the buffer solution alone was also recorded and used for blank subtraction. Melting temperature (*T*_*m*_) values are the average value of at least a pair of *T*_*m*_ values recorded during repeated melting experiments. 

The chromatographic system consisted of an Agilent 1100 Series HPLC instrument equipped with a G1311A quaternary pump, a G1379A degasser, a G1392A autosampler, a G1315B photodiode array detector furnished with a 13 *μ*L flow cell, and an Agilent ChemStation for data acquisition and analysis (Rev. A 10.02), all of them from Agilent Technologies (Waldbronn, Germany). The temperature was controlled using an HPLC column heater GECKO 2000 (CIL, France). A Phenomenex BioSep-SEC-S 2000 column (300 × 7.8 mm, particle size of 5 *μ*m, and pore of size 145 Å) from Phenomenex (Torrance, CA, USA) was used for the chromatographic separation. The mobile phase was a pH 7.1 buffer (0.1 M potassium phosphate). The flow rate was set to 1.0 mL/min. A volume of 15 *μ*L of the sample (5 *μ*M in DNA) was injected, and the temperature was set to 20, 30, and 40°C. Absorbance spectra were recorded between 200 and 500 nm.

### 2.3. Data Analysis

In this study, the analysis of multivariate data recorded along the melting experiments was done using house-made software based on the implementation of the van't Hoff equation [15].

Spectra recorded along melting experiments were arranged in a table or data matrix, whose size was *m* rows (spectra recorded) and *n* columns (wavelengths measured).

The goal of the data analysis was to calculate the distribution diagrams and pure (individual) spectra for all the *nc* spectroscopically active species considered throughout the experiment. The distribution diagram provides information about the thermodynamics of the melting processes. In addition, the shape and intensity of the pure spectra may provide qualitative information about the structure of the species.

With this goal in mind, data matrix **D** was decomposed according to Beer-Lambert-Bouguer's law in matrix form:

(1) $$\begin{array}{c} D=CS^{T}+E, \end{array}$$

where **C** is the matrix (*m* × *nc*) containing the distribution diagram, **S** ^**T**^ is the matrix (*nc* × *n*) containing the pure spectra, and **E** is the matrix of data (*m* × *n*) not explained by the proposed decomposition.

The mathematical decomposition of **D** into matrices **C**, **S** ^**T**^, and **E** may be done basically in two different ways, depending on whether a physicochemical model is initially proposed (hard-modeling approach) or not (soft-modeling approach). In this work, a hard-modeling approach has been applied. In this case, the physicochemical model is related to the thermodynamics of DNA unfolding. Hence, for the unfolding of intramolecular structures such as those studied here, the chemical equation and the corresponding equilibrium constant may be written as

(2) $$\begin{array}{c} \text{DN}{\text{A}}_{\text{folded}}+\text{heat}⟷\text{DN}{\text{A}}_{\text{unfolded}},\\ {\text{K}}_{\text{unfolding}}=\frac{\left[\text{DN}{\text{A}}_{\text{ unfolded}}\right]}{\left[\text{DN}{\text{A}}_{\text{ folded}}\right]}. \end{array}$$

The concentration of the folded and unfolded forms is temperature-dependent. Accordingly, the equilibrium constant depends on temperature according to the van't Hoff equation:

(3) $$\begin{array}{c} \ln K_{\text{unfolding}}=-\frac{\Delta H_{vH}}{RT}+\frac{\Delta S_{vH}}{R}. \end{array}$$

In this case, it is assumed that Δ*H*_*vH*_ and Δ*S*_*vH*_ will not change throughout the range of temperatures studied here. Whenever a physicochemical model is applied, the distribution diagram in **C** complies with the proposed model. Accordingly, the proposed values for the equilibrium constants and the shape of the pure spectra in **S** ^**T**^ are refined to explain satisfactorily data in **D**, whereas residuals in **E** are minimized. Hard modeling of melting experiments was done with a modified version of the MCR-ALS procedure that includes the model proposed in (3) for the unfolding of intramolecular structures. 

## 3. Results and Discussion

**Figure 2:**
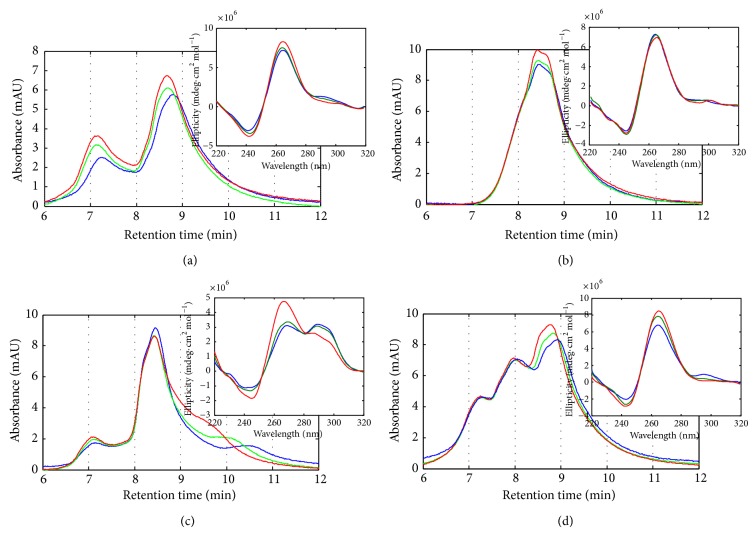
SEC profiles and CD spectra recorded for ckit21 (a), ckit21T (b), ckit21T18 (c), and ckit21T21 (d) at 21 (blue), 30 (green), and 40°C (red) and pH 7.1.

Figure 2 shows the SEC chromatograms and CD spectra (insets) recorded for all four sequences at 20, 30, and 40°C. Higher temperatures could not be achieved due to the limitations of the chromatographic column. DNA samples were prepared by direct dilution from a stock solution, heated at 95°C for 10 minutes, and slowly cooled overnight before measurements were done.

The shape of the CD spectrum of a ckit21 sample measured at pH 7 and 20°C showed the typical features related to parallel G-quadruplexes, like a large positive band around 265 nm and a negative band around 240 nm. The small shoulder at 295 nm may reflect a certain contribution of antiparallel structure. The CD spectrum of ckit21T, on the contrary, did not show any contribution at 295 nm, reflecting a clear parallel conformation. The CD spectrum of ckit21T18 at pH 7 and 25°C was clearly different from that measured for ckit21, because it showed two large positive bands around 265 and 295 nm, which denoted a mixed antiparallel/parallel topology in the same molecule or the presence of a mixture of structures in different molecules. Finally, the CD spectrum of ckit21T21 was similar to that measured for ckit21 as it contained a large positive band centered at 265 nm, which denoted a major parallel topology, and a small shoulder at 295 nm.

**Figure 3:**
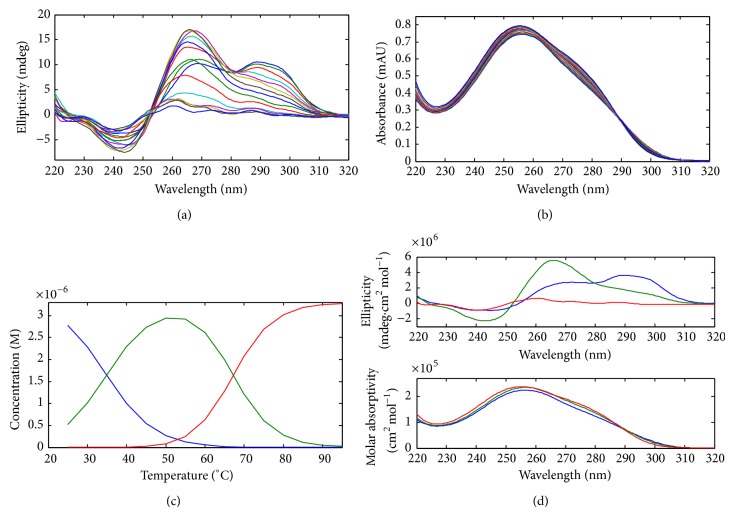
Melting experiment of ckit21T18 monitored simultaneously by CD and molecular absorption spectroscopies. (a) Experimental CD spectra. (b) Experimental molecular absorption spectra. (c) Calculated distribution diagram. (d) Calculated pure CD and molecular absorption spectra. Concentration of DNA equal to 3.3 microM, pH 7.1, and 150 mM ionic strength.

Melting experiments simultaneously monitored with CD and molecular absorption spectroscopies were carried out to evaluate the relative stability of the structures formed by the ckit21-based sequences. Analysis of the whole set of spectra recorded along the melting experiments was done by means of a multivariate data analysis method [15]. The number of species present along the melting process, as well as the associated thermodynamic parameters, (*T*_*m*_ and changes in enthalpy and entropy) was determined in this way. Upon heating, all four G-quadruplex structures unfolded with *T*_*m*_ values equal to 74°C (ckit21), 82°C (ckit21T), 66°C (ckit21T18), and 71°C (ckit21T21). The unfolding of the ckit21T18 clearly took place in two steps (Figure 3). Upon heating, the band at 295 nm was completely lost at 55°C, whereas the intensity of the band at 265 nm increased dramatically. At this point, the parallel structure was clearly the major one. Finally, this structure was lost after heating up to 90°C. The determined value of *T*_*m*_ was 66 ± 1°C, which was clearly lower than for ckit21. It seems that the existence of a two-base third loop produced the shift of the parallel to antiparallel equilibrium proposed by Hsu et al. to this last position [6]. Thermodynamic parameters calculated from melting experiments are reported in Table 1. The native sequence showed the highest values for the thermodynamic parameters, which reflected the higher stability of this sequence in front of the mutated ones. The standard change in free energy comes from the high values of standard changes both in enthalpy and in entropy. On the contrary, the ckit21T18 sequence showed the lowest values, whereas ckit21T21 showed intermediate values. From the data shown in this table, it was clear that the native sequence was the most stable at 37°C. Also, it seems that the G18T mutation reduced dramatically the stability of the structure. It is known that the formation of a tetrad implies the release of about 15–25 kcal/mol, depending on factors such as a stacking of additional bases on the tetrad. In our case, unfolding of the ckit21 sequence required a change in enthalpy per tetrad of 17 kcal/mol. Overall, unfolding of this sequence required around 6 kcal/mol more than those of the mutated sequences ckit21T18 and ckit21T21. This difference could be due to the additional stacking of the terminal guanine at the 3′ end in the wild sequence. In the case of ckit21T21, the limited contribution of the terminal thymine bases to the overall stability of quadruplex structures had been already pointed out [1].

CD spectroscopy is a low-resolution technique which does not allow discerning completely the presence of mixtures or mixed conformations. On the other hand, the recorded SEC chromatograms may reveal clear differences among the conformational space spanned by the four different sequences. Overall, SEC results allowed a qualitative correlation with the results obtained from spectroscopy.

**Figure 4:**
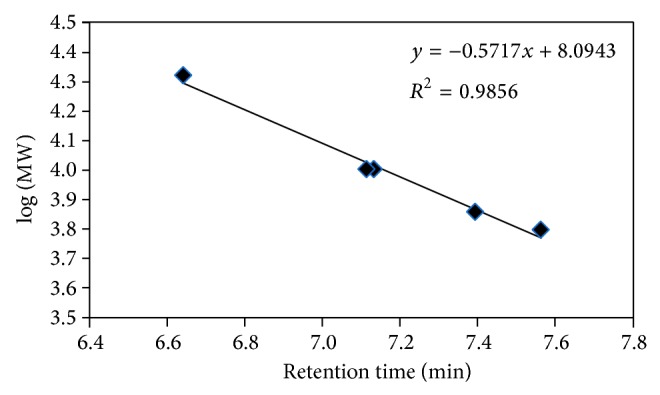
Calibration of chromatographic system. The oligonucleotides injected are 5′-T_21_-3′ (MW: 6326.2 g·mol^−1^), 5′-T_24_-3′ (MW: 7238.7 g·mol^−1^), 5′-ACC CCC TGC ATC TGC ATG CCC CCT CCC ACC CCC T-3′ (MW: 10,057.5 g·mol^−1^), 5′-ACC CCC TGC ATC TTT TTG CCC CCT CCC ACC CCC T-3′ (MW: 10,076.5 g·mol^−1^), and the Watson-Crick duplex formed by 5′-ACC CCC TGC ATC TGC ATG CCC CCT CCC ACC CCC T-3′ and 5′-AGG GGG TGG GAG GGG GCA TGC AGA TGC AGG GGG T-3′ (MW: 20,872.7 g·mol^−1^). All of these sequences form lineal structures at pH 7.1 and 20°C.

The chromatogram of the ckitG4 was the simplest among the four considered, because the recorded peak at 8.5 minutes is almost Gaussian-shaped and it lacks minor peaks at lower and higher elution times than the principal peak. Only a shoulder may be glimpsed at around 8 minutes. According to the SEC calibration with lineal structures (Figure 4), the linear structure of ckit21T should be eluted at around 7.5 minutes. Accordingly, the main peak at 8.5 minutes was related to a folded structure whose hydrodynamic volume was smaller than that corresponding to the linear structure. According to the CD spectrum, the folded structure was related to a parallel G-quadruplex. The presence of a single almost-Gaussian SEC band was correlated with the clear parallel G-quadruplex conformation detected by CD. In the temperature range tested, no clear changes were observable.

In contrast to ckit21T, the wild ckit21 sequence showed a more complex conformational space, reflected in the presence of two main peaks at 7.2 and 8.8 minutes. As before, the peak at 8.6 minutes was related to the parallel G-quadruplex. On the other hand, the peak at 7.2 minutes may be associated with some kind of multimeric species, like dimers [16]. The mutation of cytosine and adenine by thymine bases in ckit21T seems to prevent the formation of such aggregates. As reflected in the CD spectra, the shape of the SEC chromatograms hardly changes upon heating.

Concomitant with the features observed in the CD spectrum at 20°C, the chromatogram of ckit21T18 was the one that showed the most striking differences in relation to the other sequences. The chromatogram showed a large peak at 8.5 minutes and two small peaks at 7.1 and 10.5 minutes. According to the previous results, these peaks were related to the parallel G-quadruplex, aggregates, and the antiparallel conformation, respectively. Upon heating, the peak at 10.5 minutes slowly disappeared to merge with that corresponding to the G-quadruplex. This behavior was concomitant with that observed in CD spectroscopy and was related to the shift of the conformational equilibrium to the parallel conformation. Based on the SEC results, it seems that the CD spectrum at 20°C is related to a mixture of species showing parallel and antiparallel conformations, rather than with a unique species showing a mixed conformation.

Finally, the chromatogram of ckit21T21 showed three main peaks at 7.2, 8.1, and 8.9 minutes. This profile was qualitatively similar to that of the wild sequence, which again was in accordance with both CD and melting experiments. Only the presence of a peak at 8.1 minutes makes the difference between both sequences. These results agree qualitatively with those obtained at higher concentration of DNA [8].

## 4. Conclusions

Several conclusions may be drawn from the results shown here. Firstly, the utility of variable-temperature SEC chromatography as a useful tool to study the conformational equilibria of G-quadruplexes has been shown. The chromatographic profiles may be qualitatively related to those obtained by means of other techniques, such as CD. Secondly, mutation of bases at the loops produces dramatic changes in the conformational equilibria. The assumption that four guanine tracts of equal length produce conformational homogeneity may not be true and must be checked by means of complementary techniques, such as SEC. 

## Figures and Tables

**Table 1:** Thermodynamic parameters related to the unfolding of the three sequences studied in this work. Experiments have been performed at least three times. Parameters are done in terms of mean value and standard deviation.

Sequence	Δ*H* ^0^ (kcal·mol^−1^)	Δ*S* ^0^ (cal·K^−1^·mol^−1^)	Δ*G* ^0^ (kcal·mol^−1^) at 37°C	*T*_*m*_ (°C)	Reference
ckit21	51 ± 3	145 ± 9	5.4 ± 0.6	74 ± 1	This work
ckit21T	46	130	5.8	82	[12]
ckit21T18	44 ± 2	131 ± 3	3.8 ± 0.4	66 ± 2	This work
ckit21T21	46 ± 4	135 ± 12	4.5 ± 0.4	71 ± 1	This work
